# Sex-related disparities in the incidence and outcomes of hemorrhagic stroke among type 2 diabetes patients: a propensity score matching analysis using the Spanish National Hospital Discharge Database for the period 2016–18

**DOI:** 10.1186/s12933-021-01334-2

**Published:** 2021-07-09

**Authors:** Ana Lopez-de-Andres, Rodrigo Jimenez-Garcia, Valentín Hernández-Barrera, Isabel Jiménez-Trujillo, José M. de Miguel-Yanes, David Carabantes-Alarcon, Javier de Miguel-Diez, Marta Lopez-Herranz

**Affiliations:** 1 Department of Public Health & Maternal and Child Health, Faculty of Medicine, Universidad Complutense de Madrid, 28040 Madrid, Spain; 2 Preventive Medicine and Public Health Teaching and Research Unit, Health Sciences Faculty, Rey Juan Carlos University, Alcorcón, Madrid, Spain; 3 Internal Medicine Department. Hospital General, Universitario Gregorio Marañón, Universidad Complutense de Madrid, Instituto de Investigación Sanitaria Gregorio Marañón (IiSGM), Madrid, Spain; 4 Respiratory Care Department, Hospital General Universitario Gregorio Marañón, Universidad Complutense de Madrid, Instituto de Investigación Sanitaria Gregorio Marañón (IiSGM), Madrid, Spain; 5 Faculty of Nursing, Physiotherapy and Podology, Universidad Complutense de Madrid, Madrid, Spain

**Keywords:** Hemorrhagic stroke, Type 2 diabetes mellitus, Sex differences, Incidence, In-hospital mortality, Oral anticoagulants, Antiplatelet agents

## Abstract

**Background:**

To analyze incidence, use of therapeutic procedures, use of oral anticoagulants (OACs) and antiplatelet agents prior to hospitalization, and in-hospital outcomes among patients who were hospitalized with hemorrhagic stroke (HS) according to the presence of type 2 diabetes mellitus (T2DM) in Spain (2016–2018) and to assess the role of sex differences among those with T2DM.

**Methods:**

Using the Spanish National Hospital Discharge Database we estimated the incidence of HS hospitalizations in men and women aged ≥ 35 years with and without T2DM. Propensity score matching (PSM) was used to compare population subgroups according to sex and the presence of T2DM.

**Results:**

HS was coded in 31,425 men and 24,975 women, of whom 11,915 (21.12%) had T2DM. The adjusted incidence of HS was significantly higher in patients with T2DM (both sexes) than in non-T2DM individuals (IRR 1.15; 95% CI 1.12–1.17). The incidence of HS was higher in men with T2DM than in T2DM women (adjusted IRR 1.60; 95% CI 1.57–1.63). After PSM, men and women with T2DM have significantly less frequently received decompressive craniectomy than those without T2DM. In-hospital mortality (IHM) was higher among T2DM women than matched non-T2DM women (32.89% vs 30.83%; p = 0.037), with no differences among men. Decompressive craniectomy was significantly more common in men than in matched women with T2DM (5.81% vs. 3.33%; p < 0.001). IHM was higher among T2DM women than T2DM men (32.89% vs. 28.28%; p < 0.001). After adjusting for confounders with multivariable logistic regression, women with T2DM had a 18% higher mortality risk than T2DM men (OR 1.18; 95% CI 1.07–1.29). Use of OACs and antiplatelet agents prior to hospitalization were associated to higher IHM in men and women with and without T2DM.

**Conclusions:**

T2DM is associated with a higher incidence of HS and with less frequent use of decompressive craniectomy in both sexes, but with higher IHM only among women. Sex differences were detected in T2DM patients who had experienced HS, with higher incidence rates, more frequent decompressive craniectomy, and lower IHM in men than in women.

**Supplementary Information:**

The online version contains supplementary material available at 10.1186/s12933-021-01334-2.

## Background

Diabetes is a major independent risk factor for neurovascular disease [1] that is common in patients who have experienced ischemic stroke, with a prevalence of approximately 20–33% [2–4]. Compared with non-diabetic patients, diabetic patients have poor functional outcome, worse long-term vascular prognosis, and higher mortality after stroke onset [5]. However, given the inconsistent associations found in previous investigations, no consensus has been reached on the role of diabetes in the occurrence of hemorrhagic stroke (HS) [6–11]. Some studies reported no significant relationship between diabetes and HS [7, 8], while others showed a significant and positive association [9, 10] or found that HS occurs significantly less frequently in patients with diabetes [11].

Sex differences may play an active role in the incidence and outcomes of stroke among persons with diabetes [12–14]. Price et al. [14] concluded that among women, diabetes was associated with a slightly but significantly increased risk of intracerebral hemorrhage (ICH) (adjusted RR 1.31; 95% CI 1.04–1.65) and substantially reduced the risk for subarachnoid hemorrhage (SAH) (adjusted RR 0.43; 95% CI 0.26–0.69). However other studies have reported contradictory results regarding sex differences in the risk of stroke associated with diabetes [15, 16]. A UK study including nearly 2 million individuals found no evidence to suggest that the association between diabetes and stroke subtypes differed between women and men [15]. Population-based studies in Spain showed that women with type 2 diabetes (T2DM) less frequently underwent therapeutic procedures and had poorer outcomes (higher in-hospital mortality [IHM] and readmission rates) than diabetic men, although no statistical differences were found for either sex when comparing T2DM and non-diabetic patients admitted for HS [8, 17]. In their analysis of long-term outcomes, Szlachetka et al. [9] found non-significant differences in the risk of HS mortality when T2DM women were compared with T2DM men.

Differences in the prevalence of comorbidities and in the use of oral anticoagulants (OACs) and antiplatelet agents according to the presence of T2DM and sex may explain these inconclusive findings [6, 8, 9, 17–23].

For all the previous reasons we used administrative data from an entire country over a three-year period to compare incidence, clinical characteristics, use of therapeutic procedures, use of OACs and antiplatelet agents, and in-hospital outcomes in patients hospitalized with a primary diagnosis of HS according to T2DM and sex. We used propensity score matching (PSM) to compare in-hospital outcomes after HS between men and women with and without T2DM, and between men and women with T2DM. Finally, we identified the variables associated with IHM for patients with T2DM according to sex.

## Methods

### Design, setting, and participants

We performed a retrospective, epidemiological, observational study based on hospital discharge reports collected through the Spanish National Hospital Discharge Database (SNHDD) for the period running from 1 January 2016 to 31 December 2018.

All Spanish regions send their local discharge records to the Spanish Ministry of Health. Every year, data from over 4 million individuals admitted to private and public hospitals are recorded, covering over 95% of all admissions. The discharge records are coded based on the International Classification of Disease, Tenth Revision (ICD-10). The database provides up to 20 diagnoses and 20 procedures for each hospitalization. More details on this database are available online [24].

### Study population

We selected patients aged ≥ 35 years with a primary diagnosis of HS using the specific ICD-10 codes (Additional file 1: Table S1) recorded in the discharge reports. The primary diagnosis is the disease or condition that after reviewing the medical records and according to the physician completing the discharge report, is considered the main reason for the patient being hospitalized [24]. In the secondary diagnosis fields (from 2 to 20), and according to the SNHDD methodology, should only be recorded those diagnoses that have induced the use of additional therapeutic or diagnosis procedures during the hospital admission or have negatively affected the length of hospital stay (LOHS) or the IHM.

As we don’t have information on the characteristics of any previous stroke, all those patients with a code for history of previous stroke (ICD10 code Z86.73) were excluded of the study population.

The population was divided according to sex and to the presence of T2DM. Subjects with a diagnosis code for T2DM (E11.x) in any diagnosis field (i.e., positions 2–20) were classified as having T2DM.

Patients with a code for type 1 diabetes mellitus (T1DM) (E10.x) in any diagnosis field were excluded.

### Study variables

The main study variables were the incidence of HS among men and women with and without T2DM, the IHM and the LOHS in these subgroups. We also analyzed the use of decompressive craniectomy during hospitalization.

Incidences were calculated based on the Spanish population with and without T2DM grouped by age group and sex according to the Spanish National Health Survey 2017 [25].

Patient-level variables analyzed included age and sex. Comorbidity was quantified using the Charlson Comorbidity Index (CCI) calculated based on ICD-10 codes, as described elsewhere [26, 27].

Besides the diseases included in the CCI the following were specifically identified and analyzed: obesity, hypertension, lipid metabolism disorders, atrial fibrillation (AF), anemia, alcohol abuse, depression, and sepsis (See ICD-10 codes in Additional file 1: Table S1).

Concerning procedures, we studied mechanical ventilation and decompressive craniectomy. Finally, the use, prior to hospital admission, of OACs and antiplatelet agents was analyzed (Additional file 1: Table S1).

### Propensity score matching method

PSM was used to create sub-populations that were comparable according to their baseline conditions [28]. Three PSM analyses were conducted, namely, men with T2DM and non-T2DM men, women with T2DM and non-T2DM women, and T2DM men and T2DM women. The PSM analysis was conducted using multivariable logistic regression in which the matching variables were age, sex, comorbid conditions present at admission, type of HS (using up to the second digital digit in the ICD-10 codes), and year of hospitalization. These methods have been described in detail elsewhere [28, 29].

### Statistical analysis

Descriptive statistics for continuous variables were reported as mean and standard deviation or median and interquartile range (IQR); categorical variables were reported as frequency and percentage.

Incidence was analyzed using Poisson regression models adjusted for age and sex when required, providing incidence rate ratios (IRR) with 95% confidence intervals (95% CI).

Continuous variables were compared using the *t* test or Mann–Whitney test. Categorical variables were compared using the chi-square test.

McNemar’s test and a paired *t* test were used to compare study subgroups after PSM [28].

Multivariable logistic regression was the method used to identify which variables were independently associated with IHM. We constructed models separately for men and women and according to T2DM status. Finally, using the entire database, we analyzed the effect of sex. The results are shown as odds ratios (ORs) with their 95% CIs. In Additional file 1: Tables S2, S3 are shown the crude models to analyze bivariate associations of study variables with IHM. All variables with significant associations in these tables were included in the corresponding multivariable models.

The statistical analysis and PSM were conducted using Stata version 14 (Stata, College Station, Texas, USA), and significance was set at p < 0.05 (2-sided).

### Sensitivity analysis

In order to check the validity of the PSM, we also built a multivariable logistic regression model using the entire database to assess the effects of T2DM on IHM among men and women with HS after controlling for possible confounders.

### Ethical aspects

The SNHDD is owned by the Spanish Ministry of Health, which provided us with the database [30]. All personal identifiers were deleted to guarantee data confidentiality. The characteristics of this registry, which is anonymous and can be accessed upon request, obviate the need for ethics committee approval according to the Spanish legislation.

## Results

A total of 56,400 patients (55.71% men and 44.29% women) aged ≥ 35 years were hospitalized with a primary diagnosis of HS in Spain during the period 2016–2018. T2DM was diagnosed in 11,915 patients (21.12%). The prevalence of T2DM was higher among men than among women (23.68% vs. 17.90%; p < 0.001).

### Incidence of patients admitted to hospitals with a diagnosis of HS according to T2DM status

As can been seen in Table 1, the total incidence of HS was higher (p < 0.001) among the T2DM population (127.49 per 100,000 persons with T2DM) than among those without T2DM (56.82 per 100,000 persons without T2DM) resulting in an adjusted IRR of 1.15 (95% CI 1.12–1.17).

**Table 1:** Incidence of hemorrhagic stroke according to presence of type 2 diabetes mellitus, sex and age groups

Sex	Age groups	T2DM	No T2DM	p-value
		N (Inc/10^5^)	N (Inc/10^5^)	
Male	35 ~ 49 years	153 (35.01)	2556 (15.61)	< 0.001
	50 ~ 64 years	1304 (79.69)	5648 (45.4)	< 0.001
	65 ~ 79 years	3361 (158.86)	8393 (135.18)	< 0.001
	≥ 80 years	2624 (348.62)	7386 (337.89)	0.168
	All age groups	7442 (150.59)	23,983 (64.46)	< 0.001
Incidence rate ratio for men^a^ (95% CI)		1.21 (1.18–1.25)	Reference	< 0.001
Female	35 ~ 49 years	50 (14.87)	1933 (11.83)	0.110
	50 ~ 64 years	352 (38.53)	4034 (30.04)	< 0.001
	65 ~ 79 years	1671 (85.2)	6158 (81.34)	0.092
	≥ 80 years	2400 (201.23)	8377 (223.25)	< 0.001
	All age groups	4473 (101.57)	20,502 (49.9)	< 0.001
Incidence rate ratio for women^b^ (95% CI)		1.08 (1.05–1.12)	Reference	< 0.001
Total	35 ~ 49 years	203 (26.25)	4489 (13.72)	< 0.001
	50 ~ 64 years	1656 (64.94)	9682 (37.43)	< 0.001
	65 ~ 79 years	5032 (123.42)	14,551 (105.6)	< 0.001
	≥ 80 years	5024 (258.25)	15,763 (265.45)	0.089
	All age groups	11,915 (127.49)	44,485 (56.82)	< 0.001
Incidence rate ratio for both sexes^c^ (95% CI)		1.15 (1.12–1.17)	Reference	< 0.001

T2DM: Type 2 diabetes mellitus. Inc/10^5^: Incidence per 100.000 people with or without T2DM. CI Confidence interval

^a^Age adjusted incidence rate ratio for hemorrhagic stroke according to presence of type 2 diabetes mellitus among men

^b^Age adjusted incidence rate ratio for hemorrhagic stroke according to presence of type 2 diabetes mellitus among women

^c^Age and sex adjusted incidence rate ratio for hemorrhagic stroke according to presence of type 2 diabetes mellitus in the total population (both sexes)

According to sex, we found that the adjusted incidence of HS among men with T2DM was 21% higher (adjusted IRR, 1.21; 95% CI 1.18–1.25) than among non-T2DM men. The equivalent adjusted IRR for women was 1.08 (95% CI 1.05–1.12).

Men with T2DM had a higher adjusted incidence of HS than T2DM women (adjusted IRR, 1.60; 95% CI 1.57–1.63).

### Clinical characteristics and hospital outcomes for men and women admitted to hospital with a diagnosis of HS according to T2DM status

Table 2 shows clinical characteristics, therapeutic procedures, and hospital outcomes before and after PSM for men with HS according to the presence of T2DM.

**Table 2:** Clinical characteristics, use of therapeutic procedures and hospital outcomes before and after propensity score matching in men patients hospitalized with hemorrhagic stroke according to the presence of type 2 diabetes

Variables	Before PSM			After PSM		
	T2DM	No T2DM	p-value	T2DM	No T2DM	p-value
Nontraumatic subarachnoid hemorrhage, n (%)	627 (8.43)	3836 (15.99)	< 0.001	627 (8.43)	598 (8.04)	0.387
Nontraumatic intracerebral hemorrhage, n (%)	4341 (58.33)	13,225 (55.14)	< 0.001	4341 (58.33)	4372 (58.75)	0.606
Other and unspecified nontraumatic intracranial hemorrhage, n (%)	2474 (33.24)	6922 (28.86)	< 0.001	2474 (33.24)	2472 (33.22)	0.972
Age, mean (SD)	73.99 (10.7)	69.81 (14.21)	< 0.001	73.99 (10.7)	74.46 (11.39)	0.009
CCI mean (SD)	0.78 (0.89)	0.57 (0.78)	< 0.001	0.78 (0.89)	0.72 (0.88)	< 0.001
Obesity, n (%)	595 (8.00)	1026 (4.28)	< 0.001	595 (8)	511 (6.87)	0.009
Hypertension, n (%)	4936 (66.33)	12,066 (50.31)	< 0.001	4936 (66.33)	5194 (69.79)	< 0.001
Lipid metabolism disorders, n (%)	3495 (46.96)	5944 (24.78)	< 0.001	3495 (46.96)	3429 (46.08)	0.278
Renal disease, n (%)	1053 (14.15)	1553 (6.48)	< 0.001	1053 (14.15)	901 (12.11)	< 0.001
Atrial fibrillation, n (%)	1662 (22.33)	4048 (16.88)	< 0.001	1662 (22.33)	1598 (21.47)	0.205
Congestive heart failure, n (%)	427 (5.74)	806 (3.36)	< 0.001	427 (5.74)	383 (5.15)	0.112
Peripheral vascular disease, n (%)	439 (5.9)	790 (3.29)	< 0.001	439 (5.9)	404 (5.43)	0.215
Acute myocardial infarction, n (%)	385 (5.17)	699 (2.91)	< 0.001	385 (5.17)	346 (4.65)	0.139
Dementia, n (%)	357 (4.8)	1033 (4.31)	0.073	357 (4.8)	337 (4.53)	0.437
Anemia, n (%)	205 (2.75)	421 (1.76)	< 0.001	205 (2.75)	169 (2.27)	0.059
Alcohol abuse, n (%)	667 (8.96)	2280 (9.51)	0.160	667 (8.96)	601 (8.08)	0.053
Depression, n (%)	246 (3.31)	706 (2.94)	0.112	246 (3.31)	199 (2.67)	0.024
Sepsis, n (%)	114 (1.53)	343 (1.43)	0.522	114 (1.53)	93 (1.25)	0.142
Use of oral anticoagulants, n (%)	1262 (16.96)	3109 (12.96)	< 0.001	1262 (16.96)	1217 (16.35)	0.322
Use of antiplatelet agents, n (%)	1138 (15.29)	2048 (8.53)	< 0.001	1138 (15.29)	1069 (14.36)	0.111
Mechanical ventilation, n (%)	826 (11.10)	3134 (13.07)	< 0.001	826 (11.10)	820 (11.02)	0.875
Decompressive craniectomy, n (%)	441 (5.93)	1567 (6.53)	0.061	441 (5.93)	505 (6.79)	0.032
LOHS, median (IQR)	7 (11)	7 (12)	0.060	7 (11)	7 (11)	0.519
In-hospital mortality, n (%)	1968 (26.44)	5704 (23.78)	< 0.001	1968 (26.44)	1865 (25.06)	0.054

*PSM* Propensity Score Matching, *T2DM* Type 2 diabetes mellitus, *CCI* Charlson comorbidity index, *LOHS* length of hospital stay

Before matching, we found significant differences in the distribution of ICD-10 codes for HS between men with and without T2DM, with ICH being more frequent among those with T2DM, and a higher prevalence of SAH among those without T2DM. The mean age was significantly higher among men with T2DM (73.99; SD = 10.7 years) than non-T2DM men (69.81; SD = 14.21 years), and men with T2DM also had a higher mean CCI and more specific chronic conditions.

The use of OACs (16.96% vs. 12.96%; p < 0.001) and antiplatelet agents (15.29% vs. 8.53%; p < 0.001) was significantly more prevalent among men with T2DM than among those without this condition.

During hospitalization, men with T2DM received mechanical ventilation (11.10%) significantly less often than men with non-T2DM (13.07%) (p < 0.001). The median LOHS was approximately 7 days in men with T2DM and non-T2DM men. The crude IHM was 26.44% for men with T2DM and 23.78% for non-T2DM men (p < 0.001).

After PSM, rates of decompressive craniectomy were significantly lower in men with T2DM (5.93% vs. 6.79%; p = 0.032), and the difference in IHM was no longer statistically significant (26.44% vs 25.06%; p = 0.054).

The clinical characteristics, therapeutic procedures, and hospital outcomes before and after PSM for women with and without T2DM hospitalized with HS are shown in Table 3.

**Table 3:** Clinical characteristics, use of therapeutic procedures and hospital outcomes before and after propensity score matching in women patients hospitalized with hemorrhagic stroke according to the presence of type 2 diabetes

Variables	Before PSM			After PSM		
	T2DM	No T2DM	p-value	T2DM	No T2DM	p-value
Nontraumatic subarachnoid hemorrhage, n (%)	593 (13.26)	5603 (27.33)	< 0.001	593 (13.26)	607 (13.57)	0.664
Nontraumatic intracerebral hemorrhage, n (%)	2665 (59.58)	10,914 (53.23)	< 0.001	2665 (59.58)	2671 (59.71)	0.897
Other and unspecified nontraumatic intracranial hemorrhage, n (%)	1215 (27.16)	3985 (19.44)	< 0.001	1215 (27.16)	1195 (26.72)	0.633
Age, mean (SD)	78.39 (9.75)	72.33 (14.74)	< 0.001	78.39 (9.75)	79.18 (9.94)	< 0.001
CCI mean (SD)	0.67 (0.83)	0.46 (0.69)	< 0.001	0.67 (0.83)	0.61 (0.79)	0.001
Obesity, n (%)	471 (10.53)	894 (4.36)	< 0.001	471 (10.53)	380 (8.5)	0.001
Hypertension, n (%)	3079 (68.84)	10,026 (48.9)	< 0.001	3079 (68.84)	3259 (72.86)	< 0.001
Lipid metabolism disorders, n (%)	2147 (48)	5219 (25.46)	< 0.001	2147 (48)	2117 (47.33)	0.525
Renal disease, n (%)	575 (12.85)	1032 (5.03)	< 0.001	575 (12.85)	480 (10.73)	0.002
Atrial fibrillation, n (%)	1217 (27.21)	3425 (16.71)	< 0.001	1217 (27.21)	1189 (26.58)	0.504
Congestive heart failure, n (%)	322 (7.2)	697 (3.4)	< 0.001	322 (7.2)	276 (6.17)	0.052
Peripheral vascular disease, n (%)	96 (2.15)	298 (1.45)	0.001	96 (2.15)	69 (1.54)	0.034
Acute myocardial infarction, n (%)	108 (2.41)	212 (1.03)	< 0.001	108 (2.41)	98 (2.19)	0.481
Dementia, n (%)	444 (9.93)	1472 (7.18)	< 0.001	444 (9.93)	427 (9.55)	0.544
Anemia, n (%)	205 (4.58)	517 (2.52)	< 0.001	205 (4.58)	143 (3.2)	0.001
Alcohol abuse, n (%)	38 (0.85)	365 (1.78)	< 0.001	38 (0.85)	41 (0.92)	0.735
Depression, n (%)	367 (8.2)	1550 (7.56)	0.142	367 (8.2)	314 (7.02)	0.035
Sepsis, n (%)	50 (1.12)	204 (1)	0.458	50 (1.12)	27 (0.6)	0.008
Use of oral anticoagulants,, n (%)	915 (20.46)	2517 (12.28)	< 0.001	915 (20.46)	850 (19.00)	0.084
Use of antiplatelet agents, n (%)	598 (13.37)	1369 (6.68)	< 0.001	598 (13.37)	543 (12.14)	0.081
Mechanical ventilation, n (%)	426 (9.52)	2729 (13.31)	< 0.001	426 (9.52)	440 (9.84)	0.617
Decompressive craniectomy, n (%)	149 (3.33)	1064 (5.19)	< 0.001	149 (3.33)	190 (4.25)	0.023
LOHS, median (IQR)	7 (11)	8 (13)	< 0.001	7 (11)	7 (11)	0.848
In-hospital mortality, n (%)	1471 (32.89)	5830 (28.44)	< 0.001	1471 (32.89)	1379 (30.83)	0.037

*PSM* Propensity Score Matching, *T2DM* type 2 diabetes mellitus, *CCI* Charlson comorbidity index, *LOHS* length of hospital stay

Before PSM, we found significant differences in the distribution of ICD-10 codes, HS, age, and comorbidities (CCI) between women with and without T2DM, as was the case in men. However, women with T2DM had a higher prevalence of dementia than non-T2DM women (9.93% vs. 7.18%; p < 0.001) and a lower prevalence of alcohol abuse (0.85% vs. 1.78%; p < 0.001).

As found among men, more women with T2DM used OACs (20.46% vs. 12.28%; p < 0.001) and antiplatelet agents (13.37% vs. 6.68%; p < 0.001) prior to hospital admission than non-T2DM women. Women without diabetes more frequently underwent decompressive craniectomy (5.19% vs. 3.33%; p < 0.001). In addition, women with T2DM had a shorter LOHS (7 days vs. 8 days) and higher IHM (32.89% vs. 28.44%; p < 0.001) than non-T2DM women.

After PSM, decompressive craniectomy continued to be less frequent and IHM higher among T2DM women.

### Clinical characteristics and hospital outcomes for diabetic patients admitted to hospital with a diagnosis of HS according to sex

As can been seen in Table 4, when we compare T2DM men with T2DM women, we observe that men were younger (73.99 ± 9.32 years vs. 78.39 ± 9.75 years; p < 0.001), with a higher mean CCI (0.78 ± 0.69 vs. 0.67 ± 0.63). They also more frequently had kidney disease, peripheral vascular disease, and acute myocardial infarction, whereas women more frequently had obesity, hypertension, AF, congestive heart failure, dementia, anemia, and depression. Furthermore, alcohol abuse was more prevalent in men than in women (8.96% vs. 0.85%; p < 0.001). Regarding the use of OACs, it was higher among women (20.46% vs. 16.96%; p < 0.001), whereas the use of antiplatelet among men (15.29% vs. 6.68%; p = 0.004).

**Table 4:** Clinical characteristics, use of therapeutic procedures and hospital outcomes before and after propensity score matching among men and women with type2 diabetes hospitalized with hemorrhagic stroke

Variables	BEFORE PSM			AFTER PSM		
	T2DM Men	T2DM women	p-value	T2DM Men	T2DM women	p-value
35–49 years, n (%)	153 (2.06)	50 (1.12)	< 0.001	33 (0.74)	50 (1.12)	0.061
50–64 years, n (%)	1304 (17.52)	352 (7.87)	< 0.001	387 (8.65)	352 (7.87)	0.179
65–79 years, n (%)	3361 (45.16)	1671 (37.36)	< 0.001	1785 (39.91)	1671 (37.36)	0.059
≥ 80 years, n (%)	2624 (35.26)	2400 (53.66)	< 0.001	2268 (50.7)	2400 (53.66)	0.005
Age, mean (SD)	73.99 (9.32)	78.39 (9.75)	< 0.001	77.87 (9.32)	78.39 (9.75)	0.010
CCI mean (SD)	0.78 (0.69)	0.67 (0.63)	0.001	0.72 (0.62)	0.67 (0.63)	0.004
Obesity, n (%)	595 (8)	471 (10.53)	< 0.001	407 (9.1)	471 (10.53)	0.023
Hypertension, n (%)	4936 (66.33)	3079 (68.84)	0.005	3074 (68.72)	3079 (68.84)	0.909
Lipid metabolism disorders, n (%)	3495 (46.96)	2147 (48)	0.273	2119 (47.37)	2147 (48)	0.553
Renal disease, n (%)	1053 (14.15)	575 (12.85)	0.046	547 (12.23)	575 (12.85)	0.371
Atrial fibrillation, n (%)	1662 (22.33)	1217 (27.21)	< 0.001	1198 (26.78)	1217 (27.21)	0.651
Congestive heart failure, n (%)	427 (5.74)	322 (7.2)	0.001	300 (6.71)	322 (7.2)	0.360
Peripheral vascular disease, n (%)	439 (5.9)	96 (2.15)	< 0.001	99 (2.21)	96 (2.15)	0.828
Acute myocardial infarction, n (%)	385 (5.17)	108 (2.41)	< 0.001	109 (2.44)	108 (2.41)	0.945
Dementia, n (%)	357 (4.8)	444 (9.93)	< 0.001	316 (7.06)	444 (9.93)	< 0.001
Anemia, n (%)	205 (2.75)	205 (4.58)	< 0.001	141 (3.15)	205 (4.58)	< 0.001
Alcohol abuse, n (%)	667 (8.96)	38 (0.85)	< 0.001	308 (6.89)	38 (0.85)	< 0.001
Depression, n (%)	246 (3.31)	367 (8.2)	< 0.001	228 (5.1)	367 (8.2)	< 0.001
Sepsis, n (%)	114 (1.53)	50 (1.12)	0.060	55 (1.23)	50 (1.12)	0.624
Use of oral anticoagulants, n (%)	1262 (16.96)	915 (20.46)	< 0.001	871 (19.47)	915 (20.46)	0.244
Use of antiplatelet agents, n (%)	1138 (15.29)	598 (13.37)	0.004	627 (14.02)	598 (13.37)	0.372
Mechanical ventilation, n (%)	826 (11.10)	426 (9.52)	0.007	418 (9.34)	426 (9.52)	0.772
Decompressive craniectomy, n (%)	441 (5.93)	149 (3.33)	< 0.001	260 (5.81)	149 (3.33)	< 0.001
LOHS, median (IQR)	7 (11)	7 (11)	0.627	7 (11)	7 (11)	0.764
In-hospital mortality, n (%)	1968 (26.44)	1471 (32.89)	< 0.001	1265 (28.28)	1471 (32.89)	< 0.001

*T2DM* type 2 diabetes mellitus, *CCI* Charlson comorbidity index, *LOHS* length of hospital stay

After PSM, men with T2DM underwent decompressive craniectomy significantly more frequently than women with T2DM (5.81% vs. 3.33%; p < 0.001), and IHM was 3.5% higher among T2DM women than T2DM men (32.89% vs. 28.28%; p < 0.001).

### Multivariable analysis of variables associated with IHM among diabetic men and women with HS

In Table 5 are shown the results of the multivariable logistic regression models to identify those variables independently associated with IHM. The results of the crude analysis are shown in Additional file 1: Table S2. After multivariable adjustment**,** the risk of dying in hospital increased with age, sepsis, use of OACs, use of antiplatelet agents and the need for mechanical ventilation during admission among men and women with T2DM. However, renal disease and dementia were the only factors associated with IHM in men with T2DM.

**Table 5:** Multivariable logistic regression analysis of factors associated with in hospital mortality among diabetic patients with hemorrhagic stroke according to sex

Variables	Male	Female	Both
	OR (95% CI)	OR (95% CI)	OR (95% CI)
35-49 years	1	1	1
50-64 years	NS	NS	NS
65-79 years	2.16 (1.32–3.48)	1.97 (1.02–4.29)	2.01 (1.34–3.02)
≥ 80 years	3.88 (2.69–6.12)	3.62 (1.67–7.91)	3.69 (2.47–5.52)
Renal disease	1.60 (1.39–191)	NS	1.40 (1.25–1.60)
Dementia	1.66 (1.30–2.25)	NS	1.34 (1.09–1.54)
Sepsis	3.81 (243–5.82)	3.13 (1.65–5.92)	3.62 (2.51–5.13)
Use of oral anticoagulants	1.52 (1.32–1.74)	1.54 (1.31–1.77)	1.53 (1.35–1.70)
Use of antiplatelet agents	1.23 (1.01–1.44)	1.27 (1.02–154)	1.25 (1.05–1.38)
Mechanical ventilation	12.64 (10.55–15.10)	9.53 (7.40–12.25)	11.60 (9.99–13.41)
Decompressive craniectomy	0.27 (0.19–0.36)	0.34 (0.22–0.57)	0.30 (0.24–0.39)
Female sex	NA	NA	1.18 (1.07–1.29)

*T2DM* type 2 diabetes mellitus, *NA* not available, *NS* not significant

In Additional file 1: Tables 2 are shown the crude models to analyze bivariate associations of study variables with IHM. All variables with significant associations in this table were included in the corresponding multivariable models. Only variables with significant results in the multivariable regression in any of the three models are shown in the table

Undergoing decompressive craniectomy reduced IHM in T2DM patients irrespective of sex (OR 0.27; 95% CI 0.19–0.36 in men; OR 0.34; 95% CI 0.22–0.57 in women).

We found that women with T2DM were significantly more likely to die in hospital than T2DM men (OR 1.18; 95% CI 1.07–1.29).

Finally, the results of the crude and multivariable sensitivity analyses (Additional file 1: Table S3 and Table 4 respectively) confirm those obtained with PSM. No differences were found in the IHM rate according to diabetes status for men (OR 1.08; 95% CI 0.99–1.18), and the probability of dying for women who had T2DM was 12% higher (OR 1.12; 95% CI 1.02–1.24) than for non-diabetic women.

## Discussion

This nationwide registry and population-based observational study showed that incidence rates of HS were higher in patients with T2DM than in those without T2DM in all age groups analysed, except in those aged 80 years or over. After PSM, decompressive craniectomy was used less frequently in T2DM patients than in non-T2DM patients and less frequently in T2DM women than in T2DM men. IHM was significantly higher in women with T2DM than in non-diabetic women. Decompressive craniectomy appeared to be associated with a lower IHM among T2DM patients. In the fully adjusted model, women with T2DM had a 18% higher adjusted risk of dying in hospital after HS than men with T2DM.

According to our database, the incidence of HS was higher in patients with diabetes than in those without diabetes, irrespective of sex. This finding has been reported elsewhere [8, 10, 31, 32]. Furthermore, incidence rates of HS were higher in T2DM men than in T2DM women. These trends are in accordance with data from diabetic populations [8, 12, 31]. Although the American Heart Association/American Stroke Association has summarized the particularities of HS in women in a separate document owing to the increasing weight of stroke-induced mortality in women [33], virtually all studies show a persistently higher incidence of HS in men. What is striking is that these higher rates cannot be completely explained based on established risk factors [34, 35]. The results of the present study are in line with those found in the literature, thereby demonstrating that patients with diabetes have a poorer risk profile in the presence of concomitant risk factors than patients without diabetes and that they are also more often affected by comorbidities [9, 10]. It has been reported that the long-term mortality rate in ICH patients with T2DM is 2.32-fold higher than in those without diabetes [36]. In addition, as expected, and consistent with other investigations, older age, use of OACs and antiplatelet agents, requiring mechanical ventilation, and sepsis were risk factors for IHM in all subgroups [6, 8, 9, 17–23, 37].

In our investigation patients with T2DM used OACs and antiplatelet agents prior to HS in a higher proportion than non-T2DM patients as has been reported by other authors [18, 22]. The higher prevalence of all cardiovascular conditions analyzed among those with diabetes, especially AF, can explain this association [18, 22].

We agree with many other observational and clinical studies finding increased IHM after HS among those taking OACs, warfarin and non–vitamin K antagonist oral anticoagulants (NOACs) and antiplatelet agents prior to hospitalization [18–23].

Xian et al. conducted and observational study with 219, 701 patients who suffered HS and found an IHM of 22.6% for those not taking OACs, 27.0% for those taking NOACs and 32.8% for those with warfarin. The multivariable regression showed that, compared to those not taking OACs, the adjusted odds ratio for IHM was 1.27 (95% CI 1.20- 1.34; P < 0.001) for NOACs and 1.67 (95% CI 1.60–1.74; P < 0.001) for warfarin [18].

Very similar figures were reported by Inohara et al. among 141, 311 patients with HS who reported that after adjustment for confounders, prior use of warfarin (OR 1.62; [97.5% CI 1.53 to 1.71) and prior use of NOACs (OR, 1.21 [97.5% CI 1.11 to 1.32]) were associated with increased odds of IHM as compared with no prior use of OACs. These findings were consistent when confined to patients without preceding use of any antiplatelet agents [22].

Data on 13,291 patients with ICH collected by the Swedish Stroke Register between 2012 and 2016 showed that OACs and antiplatelet were associated with higher risk of early mortality (≤ 24 h): with a hazard ratio of 1.93 (95% CI 1.57–2.38) for OACs and 1.32 (95% CI 1.13–1.54) for antiplatelet agents [21].

A meta-analysis including data from 25 cohorts, reported higher IHM in patients with preceding use of antiplatelet therapy in both univariate (OR 1.41, 95% CI1.21–1.64) and multivariable-adjusted pooled analyses (OR 1.27,95 CI 1.10 to 1.47) [23].

The negative effect of OACs and antiplatelet agents is likely related to a larger baseline hematoma and increased risk of hematoma expansion [18–23].

The volume, location and the presence of hematoma expansion were not available in the SNHDD which may explain the differences in IHM between patients with or without OACs and antiplatelet therapy. The lack of this information in administrative databases was also reported by Inohara et al. who argued that because harder end points, such as in-hospital death, given that the hematoma volume and expansion may be caused by the preceding use of NOACs or warfarin resulting in the worse clinical outcomes, including these variables in the models would not be appropriate [22].

Unfortunately, in the SNHDD, like in databases used in most observational studies, we cannot differentiate the type or number of OACs and antiplatelet agents used, the duration of treatment, and when the last dose was taken [18–23].

However, the worse functional outcome and death in patients taking these medications may be confounded by not totally controlled adverse prognostic factors such as older age and higher prevalence or severity of vascular disease [18–23, 38]. In our investigation crude data analysis showed that AF was an independent predictor for IHM (Additional file 1: Table S3), However, after multivariable adjustment this association become not significant while OACs and antiplatelet agents remained in the model. Several authors agreed with us finding that AF was a risk factor in the unadjusted model but showed no independent effect on mortality after adjustment for antithrombotic or anticoagulant pretreatment [39, 40]. AF is the most frequent condition for indication of OACs and therefore is logical that only one of the variables remains significant [19, 40]. Further investigation is required to clarify this point.

Mechanical ventilation, too, is a well-known risk factor for mortality after HS in patients with T2DM [41].

The results of the present study indicate that during admission for HS, men and women with T2DM undergo decompressive craniectomy less frequently than matched non-T2DM men and women. The underuse of decompressive craniectomy among people with diabetes has been described previously [8]. Furthermore, this procedure was less used in T2DM women than in T2DM men. Lower decompressive craniectomy rates in women may be explained by the fact that women with stroke have more in-hospital complications than men, and this may contribute to treatment decisions that involve a less invasive approach [35]. Decompressive craniectomy has proven to be beneficial in terms of survival and even functional outcome in patients with middle cerebral artery infarction; however, the benefit for patients with spontaneous ICH is still debatable. A recent systematic review showed that decompressive craniectomy reduced mortality (RR, 0.67; 95% CI 0.53–0.85; p = 0.0008) and might even improve functional outcome in patients with spontaneous ICH [42].

Another possible reason for the less frequent use of this procedure is that several studies show diabetes to independently predict worse functional outcomes after decompressive craniectomy [43, 44]. Tian et al. [44] found that diabetes was associated with unfavorable discharge outcomes after decompressive craniectomy (OR, 0.29; 95% CI 0.10–0.81; p = 0.018). However, we found that decompressive craniectomy was strongly associated with a lower IHM in patients with diabetes, thus making it necessary to investigate the reasons for these differences. If possible, future investigations should control the severity of stroke at baseline when the indication of decompressive craniectomy is assessed.

Men and women with diabetes admitted with HS have higher IHM than men and women without diabetes. This finding confirms those of previous research in Spain from 2003 to 2012, albeit without PSM, where the IHM reported was higher in patients with T2DM than in patients without T2DM admitted for HS (32.36% vs. 29.22%) [8]. These findings are very similar to current data. However, in men, the presence of diabetes was not an independent predictor of increased mortality in our study, as reported elsewhere [45].

The association between mortality and diabetes can be explained by the fact that diabetes induces atherosclerotic changes that cause stiffening of vessel walls, which are therefore more resistant to hemodynamic pressure, less likely to undergo dilation, and more prone to rupture [46].

During admission for HS, we found that women with diabetes had a higher IHM and that this risk remained unchanged after the multivariable regression model analysis. Investigators in the UK Prospective Diabetes Study reported that women with diabetes had more than twice the risk of men with diabetes for stroke case fatality [47]. The poorer post-stroke survival in women with diabetes than in men was also noted in the MONICA study [48]. The reason for this finding is probably multifactorial. Progression of atherosclerosis or more novel risk factors (such as markers of coagulation and inflammation, lipid peroxidation, and endothelial function) in patients with and without T2DM is more common in women than in men; therefore, women with diabetes are disadvantaged compared with men, even before their diagnosis [49]. Additionally, a UK study found the effect of diabetes to be more pronounced in women than in men with respect to markers of fibrinolysis, lipids, and blood pressure, which were mediated by greater changes in central adiposity and insulin resistance in women [50]. The cumulative effects of these disadvantages among women throughout the course of disease could explain some of the excess relative risk.

The strengths of administrative databases for HS investigation have been discussed before and include (1) reflect real-world practice at a hospital, regional, or national level, (2) are large, so rare risk factors and diseases associated with stroke can also be evaluated using databases that capture these well, (3) are less expensive and resource intensive than clinical studies, and (4) given the impracticalities of conducting regular gold standard population-based stroke incidence studies, using administrative admissions data has the potential to be a cost-effective alternative for monitoring stroke incidence and burden [51–53].

However, ICD codes can be reliably used to identify cases of stroke, only when they have been validated within both the population of interest and administrative databases that capture these codes [51, 52].

In Canada, Kokotailo et al. reported that ICD-9 and ICD-10 coding in hospital discharge databases was correct in 98% (95% CI 92–99) of patients with ICH and in 91% (95% CI 77–98) with SAH [54]. Furthermore, stroke risk factors such as diabetes mellitus, hypertension, coronary artery disease and AF were coded with high sensitivity (81% to 91%) and specificity (83% to 100%) [54].

In Taiwan’s National Health Insurance database, compared with a stroke registry, the sensitivity for acute HS was 93.1% for hospitalizations in which the primary diagnosis field contained ICD10 codes I60 or I61 [55].

According to a systematic review of validation studies of ICD codes for acute stroke hospitalizations published in 2015, the positive predictive values (PPVs) for SAH and ICH were ≥ 93% and ≥ 89%, respectively, in at least half of the studies [53]. In more recent studies from Australia, South Korea, Czech Republic and Denmark, using the primary diagnosis code only, the PPVs ranged from 85 to 91% and sensitivities from 73 to 90% for SAH. For ICH, the PPVs ranged from 75 to 92% and sensitivities from 60 to 85% [56–59].

Chang et al. compared the agreement between the attending physician’s clinical diagnosis and principal ICD-9-CM code in 85,024 patients from more than 300 hospitals in the USA. The Kappa Coefficient for ICH was 0.94 and for SAH 0.96. Factors associated with better agreement included stroke units, larger hospital size, and more severe cases [60].

Hsieh et al. concluded that case definitions with high PPV that use only the primary diagnosis may be more appropriate when it is important for researchers to identify a “pure” cohort of patients with acute HS [55].

Besides those mentioned before, specific strengths of the SNHDD are the large sample size, with data from over 56,400 episodes of HS (21.12%% with T2DM), the widespread coverage of the population of an entire country (> 95% of all hospital admissions), the standardized methodology, and the reliability of diabetes and HS coding in the SNHDD [61–63]. The validity of HS diagnosis in the SMHDD has been demonstrated before in a study conducted in a representative sample of 30 Spanish Hospitals [34] Compared with medical records the ICD 9 codes for HS as a primary diagnosis registered in the SNHDD were correct in 43 of 49 cases reviewed (87.75%) [61].

However, it is important to understand the limitations of administrative databases, as these secondary data are not collected for research purposes [51–55]. First, a particular disadvantage of administrative data is the inability to ascertain baseline stroke severity, which is an important predictor of short- and long-term outcome and therefore remains as an important residual confounder variable [51, 52, 54]. Regarding this point to assess the baseline characteristic of the HS we compared the distribution according the ICD10 codes up to the first decimal (22 categories) for patients with and without diabetes according to sex as can be seen in Additional file 1: Tables S5, S6. These tables show that for either men and women, after PSM, the distribution for almost all anatomical codes did not differ significantly by diabetes status. Second, because data are generated by routine healthcare utilization, patients with stroke who do not present to medical attention or who are misdiagnosed will not be captured. This causes a selection bias toward stroke patients having more severe and nonfatal symptoms and those who have a better access to medical coverage [52]. Third, data quality depends on two major factors: (1) documentation: the quality of data provided in the medical record and (2) coding: the quality of training/expertise of the coder and the applicability of the codes to the medical condition (s) reported in the medical record [52]. Forth, another limitation arises when codes that lead to greater reimbursement of funds to hospitals are favored over the principal reason a patient is hospitalized [51].

However, when these data are applied to an appropriate question with validated case definitions, high-quality and reliable conclusions can be inferred [52, 54]. This is confirmed by the fact that authors from many different countries have used hospital discharge administrative data to assess trends, demographic and clinical characteristics, and outcomes of hospitalizations with stroke [64–71]. In the US the National Inpatient Sample database has provided data on the economic burden, prevalence of cardiovascular risk factors and trends over time for stroke [64–66].

Authors from European countries, like Germany, Italy, France and Spain have analysed nationwide hospitalizations administrative databases to assess incidence, treatment and mortality after stroke [67–71].

Other limitations derived from the particular characteristics of the SNHDD not yet commented include the following: (1) As only those conditions or treatments that have induced the use of additional therapeutic or diagnosis procedures or negatively affected the LOHS or the IHM have to be recorded [24], the codes for insulin (ICD10 code Z79.4) or other antidiabetic medication use (ICD10 code Z79.84), which are part of the T2DM patient’s treatment and presumably don’t affect the procedures or outcomes of the hospitalization, are not codified among T2DM patients. This also affects the use of other medications such as hypertensive agents and antibiotics. However, the use of these last two medications is associated to diagnosis codes for hypertension or infections (sepsis) that have been analyzed in our investigation. (2) In the SNHDD information on laboratory results is not collected so we cannot assess the effect to hypoglycemia, hyperglycemia or glycated hemoglobin A1c levels on the IHM. These conditions have been reported as predictors of a worse outcome after HS in some but not all investigations [72–75]. (3) While the PSM process has surely helped to attenuate differences in baseline characteristics and clinical variables, complete elimination of residual confounding is difficult to achieve in observational studies. (4) Anonymity precludes the extraction of specific data that may affect the results (i.e., people who moved from one hospital to another could appear twice). (5) We did not include patients that suffered a HS during their hospitalization as this was not an objective of our investigation. However, this is an infrequent event. In Spain in the three years’ study period analysed, among those hospitalized with a primary diagnosis of myocardial infarction, only 125 HS (89/107237 among non-T2DM patients and 36/46986 among T2DM patients) that occurred after the hospital admission were diagnosed. This represents under 1 case per 1000 patients with infarction, with no significant differences between those with and without diabetes (p = 0.89). Further studies are needed to assess the effect of T2DM in the incidence of in-hospital HS. 6) We excluded all patients with a code for history of previous stroke as we intended to compare the incidence and outcomes only of the first HS. So the prevalence of recurrence stroke could not be analyzed as a factor associated with IHM. However, the proportion of men and women excluded for this reason did not differ according to sex among those with T2DM (men N = 715, 8.76% vs. women N = 454, 9.21%: p = 0.38) and without T2DM (men N = 1507, 5.91% vs. women N = 1225, 5.66%: p = 0.20). The higher prevalence of history of previous stroke among T2DM than non-T2DM patients can be explained by the higher mean age of those suffering T2DM. However, previous studies have reported that history of cerebrovascular disease are identified to a lesser degree than current strokes in administrative databases [54]. The poor coding of this risk factors may be attributable to poor charting by physicians and nursing staff, a lack of perceived importance by health technologist coders, or a lack of time to “code everything.” [54]. Therefore, the residual confounding of this and other variables commented before must be considered, and the results have to be interpreted with caution. However, in our opinion, the strengths of this study and its uniqueness clearly outweigh its limitations. To our knowledge this is the first time that an administrative database based in ICD10 from an entire country has been used to assess sex differences the incidence, characteristics and outcomes of HS according to the presence of T2DM. Furthermore, the SNHDD has been successfully used before to investigate other cardiovascular conditions in patients with T2DM [8, 17, 29, 76, 77]. In any case our study is the starting point for future investigations that should confirm or discard our conclusions using larger and more precise clinical data.

## Conclusions

In conclusion, T2DM is associated with a higher incidence of HS and with lower use of decompressive craniectomy in both sexes, although with higher IHM only among women. The sex differences between T2DM patients who experience HS take the form of higher incidence rates, more decompressive craniectomy, and lower IHM in men than in women. Use of OACs and antiplatelet agents were associated to higher IHM in men and women with and without T2DM. The use of administrative data means that we lack some relevant clinical data so the effect of residual confounding must be considered, therefore the results have to be interpreted with caution and should be confirmed in the future with clinical studies.

However, our findings should be taken into consideration when planning future actions and investigations to improve the treatment and care that T2DM patients receive. Research efforts should focus on identifying and eliminating these sex-related disparities in our health system.

## Supplementary Information

**Additional file 1.** Additional tables.

## Data Availability

According to the contract signed with the Spanish Ministry of Health and Social Services, which provided access to the databases from the Spanish National Hospital Database (*Conjunto Mínimo Basico de Datos*; CMBD), we cannot share the databases with any other investigator, and we have to destroy the databases once the investigation has concluded. Consequently, we cannot upload the databases to any public repository. However, any investigator can apply for access to the databases by filling out the questionnaire available at http://www.msssi.gob.es/estadEstudios/estadisticas/estadisticas/estMinisterio/SolicitudCMBDdocs/Formulario_Peticion_Datos_CMBD.pdf. All other relevant data are included in the paper.

## Abbreviations

AF: Atrial fibrillation
CCI: Charlson Comorbidity Index
ICD-10: International Classification of Disease, Tenth Revision
HS: Hemorrhagic stroke
IHM: In hospital mortality
ICH: Intracerebral hemorrhage
IRR: Incidence rate ratio
LOHS: Length of hospital stay
ORs: Odds ratios
PSM: Propensity score matching
SNHDD: Spanish National Hospital Discharge Database
T1DM: Type 1 diabetes mellitus
T2DM: Type 2 diabetes mellitus
