# Supplementary material for: Sex-related disparities in the incidence and outcomes of hemorrhagic stroke among type 2 diabetes patients: a propensity score matching analysis using the Spanish National Hospital Discharge Database for the period 2016–18
Source: Cardiovasc Diabetol. 2021 Jul 9;20:138. doi: 10.1186/s12933-021-01334-2 (PMC8272346; doi:10.1186/s12933-021-01334-2)
Supplement: Supplementary file 1 — Additional file 1. Additional tables. [file 12933_2021_1334_MOESM1_ESM.docx]

Table S1. International Classification of Disease 10^th^ edition (ICD-10) codes for the clinical diagnosis and procedures used in this investigation.

| **Clinical diagnosis and procedures** | **ICD-10 codes** |
| --- | --- |
| Nontraumatic subarachnoid hemorrhage | I60.0, I60.00; I60.01; I60.02; I60.1; I60.10, I60.11, I60.12, I60.2, I60.3; I60.30, I60.31, I60.32, I60.4, I60.5, I60.50, I60.51, I60.52, I60.6, I60.7, I60.8, I60.9 |
| Nontraumatic intracerebral hemorrhage | I61.0, I61.2, I61.3, I61.4, I61.5, I61.6, I61.8, I61.9 |
| Other and unspecified nontraumatic intracranial hemorrhage | I62.0, I62.00, I62.01, I62.02, I62.03, I62.1, I62.9 |
| Obesity | E66.X |
| Hypertension | I10, I16.6 |
| Lipid metabolism disorders | E78.0X-E78.5 |
| Atrial fibrillation | I48.0, I48.1, I48.2, I48.91 |
| Anemia | D50.0, D50.8, D50.9, D51.x-D53.x |
| Alcohol abuse | F10, E52, G62.1, I42.6, K29.2, K70.0, K70.3, K70.9, T51.x, Z50.2, Z71.4, Z72.1 |
| Depression | F20.4, F31.3-F31.5, F32.x, F33.x, F34.1, F41.2, F43.2 |
| Sepsis | R65.20, R65.21, A40.X, A41.XX |
| Use of oral anticoagulants | Z79.01 |
| Use of antiplatelet agents | Z79.02, Z79.82 |
| Mechanical ventilation | 5A1945Z, 5A1955Z, 5A1935Z,5A09357, 5A09457, 5A09557 |
| Decompressive craniectomy | 00J00ZZ, 00W00JZ, 00W00KZ, 0N800ZZ, 0N803ZZ, 0N804ZZ, 0NC10ZZ, 0NC13ZZ, 0NC14ZZ, 0NC30ZZ, 0NC33ZZ, 0NC34ZZ, 0NC40ZZ, 0NC43ZZ, 0NC44ZZ, 0NC50ZZ, 0NC53ZZ, 0NC54ZZ, 0NC60ZZ, 0NC63ZZ, 0NC64ZZ, 0NC70ZZ, 0NC73ZZ, 0NC74ZZ, 0NH00MZ, 0NH03MZ, 0NH04MZ, 0NP000Z, 0NP004Z, 0NP005Z, 0NP007Z, 0NP007Z, 0NP00KZ, 0NP00SZ, 0NP030Z, 0NP034Z, 0NP037Z, 0NP03KZ, 0NP03SZ, 0NP040Z, 0NP044Z, 0NP047Z, 0NP04KZ, 0NP04SZ, 0NP0X4Z, 0NP0XSZ, 0NW000Z, 0NW004Z, 0NW005Z, 0NW007Z, 0NW00JZ, 0NW00KZ, 0NW00MZ, 0NW00SZ, 0NW030Z, 0NW034Z, 0NW035Z, 0NW037Z, 0NW03JZ, 0NW03KZ, 0NW03MZ, 0NW03SZ, 0NW040Z, 0NW044Z, 0NW045Z, 0NW047Z, 0NW04JZ, 0NW04KZ, 0NW04MZ, 0NW04SZ, 0W9100Z, 0W010ZZ, 0W9130Z, 0W913ZZ, 0W9140Z, 0W914ZZ, 0WC10ZZ, 0WC13ZZ, 0WC14ZZ, 0WH10YZ, 0WH13YZ, 0WH14YZ, 0WJ10ZZ, 0WP100Z, 0WP101Z, 0WP10JZ, 0WP10YZ, 0WP130Z, 0WP131Z, 0WP13JZ, 0WP13YZ, 0WP140Z, 0WP141Z, 0WP14JZ, 0WP14HZ, 0WW00Z, 0WW101Z, 0WW103Z, 0WW10JZ, 0WW10YZ, 0WW130Z, 0WW131Z, 0WW133Z, 0WW13JZ, 0WW13YZ, 0WW140Z, 0WW141Z, 0WW143Z, 0WW14JZ, 0WW14YZ, 0N500ZZ, 0N503ZZ, 0N504ZZ, 0NB00ZZ, 0NB03ZZ, 0NB04ZZ, 0NT10ZZ, 0NT30ZZ, 0NT40ZZ, 0NT50ZZ, 0NT60ZZ, 0NT70ZZ, 009100Z, 00910ZZ, 00C10ZZ, 00C13ZZ, 00C14ZZ, 009000Z, 00900ZZ, 009030Z, 00903ZZ, 009040Z, 00904ZZ, 00C00ZZ, 00C03ZZ, 00C04ZZ, 00H003Z, 00H003Z, 00H00YZ, 00H032Z, 00H033Z, 00H03YZ, 00H042Z, 00H043Z, 00H04YZ, 00H602Z, 00H603Z, 00H60YZ, 00H632Z, 00H633Z, 00H63YZ, 00H642Z, 00H643Z, 00H64YZ, 00P000Z, 00P002Z, 00P003Z, 00P007Z, 00P00JZ, 00P00KZ, 00P00YZ, 00P030Z, 00P032Z, 00P033Z, 00P037Z, 00P03JZ, 00P03KZ, 00P03YZ, 00P040Z, 00P042Z, 00P043Z, 00P047Z, 00P04JZ, 00P04KZ, 00P04YZ, 00P600Z, 00P602Z, 00P603Z, 00P60YZ, 00P630Z, 00P632Z, 00P633Z, 00P63YZ, 00P640Z, 00P642Z, 00P643Z, 00P64YZ, 00P6X2Z, 00W000Z, 00W002Z, 00W003Z, 00W007Z, 00W00MZ, 00W00YZ, 00W030Z, 00W032Z, 00W033Z, 00W037Z, 00W03JZ, 00W03KZ, 00W03MZ, 00W03YZ, 00W040Z, 00W042Z, 00W043Z, 00W047Z, 00W04JZ, 00W04KZ, 00W04MZ, 00W04YZ, 00W600Z, 00W602Z, 00W603Z, 00W60MZ, 00W60YZ, 00W630Z, 00W632Z, 00W633Z, 00W63MZ, 00W63YZ, 00W640Z, 00W642Z, 00W643Z, 00W64MZ, 00W64YZ, 00B70ZZ, 00B73ZZ, 00B74ZZ, 0500ZZ, 00503ZZ, 00504ZZ, 00B00ZZ, 00B03ZZ, 00B04ZZ. |

Table S2. Crude analysis of factors associated with in hospital mortality among type 2 diabetic patients with hemorrhagic stroke according to sex.

|  | Male | Female | Both |
| --- | --- | --- | --- |
|  | OR (95%CI) | OR (95%CI) | OR (95%CI) |
| 35~49 years | 1 | 1 | 1 |
| 50~64 years | 1.25(0.76-2.03) | 0.81(0.35-1.85) | 1.1(0.73-1.67) |
| 65~79 years | 2.09(1.3-3.37) | 2.03(1.02-4.45) | 2.05(1.35-3.04) |
| ≥80 years | 3.79(2.34-6.15) | 3.64(1.65-8) | 3.68(2.45-5.52) |
| Obesity | 0.88(0.7-1.1) | 1.17(0.93-1.46) | 1.00(0.85-1.17) |
| Hypertension | 1.01(0.89-1.16) | 0.94(0.8-1.11) | 0.99(0.89-1.1) |
| Lipid metabolism disorders | 0.91(0.81-1.02) | 0.88(0.75-1.02) | 0.90(0.83-1.00) |
| Renal disease | 1.59(1.34-1.88) | 1.04(0.83-1.3) | 1.35(1.18-1.55) |
| Atrial fibrillation | 1.42(1.25-1.63) | 1.51(1.3-1.76) | 1.46(1.32-1.61) |
| Congestive heart failure | 1.27(1.01-1.59) | 1.07(0.82-1.38) | 1.16(0.98-1.38) |
| Peripheral vascular disease | 1.2(0.95-1.51) | 1.05(0.66-1.67) | 1.19(0.97-1.46) |
| Acute myocardial infarction | 1.03(0.8-1.33) | 0.98(0.64-1.51) | 1.03(0.83-1.27) |
| Dementia | 1.72(1.36-2.18) | 1.14(0.92-1.41) | 1.38(1.17-1.61) |
| Anemia | 0.80(0.56-1.13) | 0.88(0.63-1.21) | 0.83(0.66-1.06) |
| Alcohol abuse | 1.16(0.94-1.43) | 0.88(0.39-1.95) | 1.16(0.95-1.41) |
| Depression | 1.09(0.8-1.5) | 0.96(0.75-1.23) | 1.01(0.83-1.23) |
| Sepsis | 3.83(2.44-5.99) | 2.91(1.54-5.48) | 3.50(2.42-5.05) |
| Use of oral anticoagulants | 1.45(1.23-1.67) | 1.50(1.27-1.72) | 1.47(1.27-1.68) |
| Use of antiplatelet agents | 1.30(1.07-1.54) | 1.35(1.09-1.59) | 1.33(1.12-51) |
| Mechanical ventilation | 12.81(10.67-15.38) | 9.55(7.42-12.3) | 11.5(9.92-13.33) |
| Decompressive craniectomy | 0.26(0.19-0.35) | 0.35(0.22-0.56) | 0.28(0.22-0.37) |
| Female sex | NA | NA | 1.23(1.13-1.35) |

OR: Odds ratio. CI Confidence interval

Table S3. Crude analysis of factors associated with in hospital mortality among patients hospitalized with hemorrhagic stroke according to sex.

|  | Male | Female |
| --- | --- | --- |
|  | OR (95%CI) | OR (95%CI) |
| 35~49 years | 1 | 1 |
| 50~64 years | 1.2(0.86-1.68) | 1.02(0.55-1.89) |
| 65~79 years | 2.07(1.5-2.86) | 2.1(1.17-3.76) |
| ≥80 years | 3.93(2.83-5.46) | 4.34(2.42-7.77) |
| Obesity | 0.95(0.81-1.11) | 1.1(0.93-1.3) |
| Hypertension | 0.92(0.84-1.01) | 0.9(0.8-1.02) |
| Lipid metabolism disorders | 0.93(0.86-1.01) | 0.87(0.79-0.95) |
| Renal disease | 1.39(1.23-1.57) | 1.09(0.92-1.28) |
| Atrial fibrillation | 1.44(1.31-1.58) | 1.36(1.22-1.52) |
| Congestive heart failure | 1.37(1.16-1.61) | 1.17(0.97-1.42) |
| Peripheral vascular disease | 1.23(1.05-1.45) | 1.14(0.8-1.61) |
| Acute myocardial infarction | 1.08(0.91-1.3) | 1.22(0.91-1.65) |
| Dementia | 1.73(1.46-2.04) | 1.32(1.13-1.54) |
| Anemia | 0.78(0.6-1.01) | 0.86(0.67-1.1) |
| Alcohol abuse | 1.12(0.96-1.31) | 0.53(0.27-1.03) |
| Depression | 1.08(0.85-1.36) | 0.87(0.72-1.05) |
| Sepsis | 3.32(2.41-4.57) | 3.24(1.94-5.42) |
| Use of oral anticoagulants | 1.47(1.28-1.69) | 1.53(1.31-1.75) |
| Use of antiplatelet agents | 1.36(1.15-1.57) | 1.44(1.22-1.65) |
| Mechanical ventilation | 11.98(10.54-13.62) | 11.09(9.25-13.3) |
| Decompressive craniectomy | 0.28(0.23-0.35) | 0.23(0.17-0.33) |
| T2DM | 1.08(1.01-1.17) | 1.12(1.02-1.23) |

OR: Odds ratio. CI Confidence interval

Table S4. Logistic regression factors associated with in hospital mortality after hemorrhagic stroke among all patients and according to the presence of type 2 diabetes mellitus to assess the sex differences.

|  | **Male** | **Female** |
| --- | --- | --- |
| **Variables** | **OR (95%CI)** | **OR (95%CI)** |
| 35~49 years | 1 | 1 |
| 50~64 years | NS | BS |
| 65~79 years | 2.12(1.54-2.90) | 2.06(1.14-3.69) |
| ≥80 years | 3.81(2.76-5.31) | 4.02(2.17-7.03) |
| Renal disease | 1.52(1.31-1.72) | NA |
| Congestive heart failure | 1..23(1.02-143) | NA |
| Dementia | 1.71(1.41-2.10) | 1.20(1.01-1.43) |
| Sepsis | 3.91(2.49-4.71) | 3.47(2.11-5.73) |
| Use of oral anticoagulants | 1.61(1.32-171) | 1.63(1.39-1.84) |
| Use of antiplatelet agents | 1.33(1.12-1.55) | 1.39(1.21-1.625) |
| Mechanical ventilation | 11.99(10.68-13.77) | 11.35(9.87-14.02) |
| Decompressive craniectomy | 0.31(0.25-0.38) | 0.36(0.22-0.43) |
| T2DM | 1.10(1.01-1.21) | 1.14(1.03-1.27) |

T2DM: Type 2 diabetes mellitus. NA: Not available. NS Not significant. In Supplementary Tables 3 are shown the crude models to analyze bivariate associations of study variables with IHM. All variables with significant associations in this table were included in the corresponding multivariable models. Only variables with significant results in the multivariable regression are shown in the table. OR: Odds ratio. CI Confidence interval

Supplementary Table 5. International Classification of Disease, Tenth Revision (ICD-10) codes before and after propensity score matching in men patients hospitalized with hemorrhagic stroke as primary diagnosis according to the presence of type 2 diabetes.

| International Classification of Disease, Tenth Revision codes | BEFORE PSM | | | AFTER PSM | | |
| --- | --- | --- | --- | --- | --- | --- |
|  | No T2DM | T2DM | p-value | No T2DM | T2DM | p-value |
| I60.0 Nontraumatic subarachnoid hemorrhage from carotid siphon and bifurcation | 58(0.24) | 1(0.01) | <0.001 | 1(0.01) | 1(0.01) | 0.999 |
| I60.1 Nontraumatic subarachnoid hemorrhage from middle cerebral artery | 253(1.05) | 29(0.39) | <0.001 | 25(0.34) | 29(0.39) | 0.586 |
| I60.2 Nontraumatic subarachnoid hemorrhage from anterior communicating artery | 426(1.78) | 35(0.47) | <0.001 | 41(0.55) | 35(0.47) | 0.490 |
| I60.3 Nontraumatic subarachnoid hemorrhage from posterior communicating artery | 83(0.35) | 13(0.17) | 0.019 | 9(0.12) | 13(0.17) | 0.393 |
| I60.4 Nontraumatic subarachnoid hemorrhage from basilar artery | 56(0.23) | 13(0.17) | 0.344 | 3(0.04) | 13(0.17) | 0.012 |
| I60.5 Nontraumatic subarachnoid hemorrhage from vertebral artery | 41(0.17) | 7(0.09) | 0.137 | 4(0.05) | 7(0.09) | 0.366 |
| I60.6 Nontraumatic subarachnoid hemorrhage from other intracranial arteries | 132(0.55) | 28(0.38) | 0.065 | 13(0.17) | 28(0.38) | 0.019 |
| I60.7 Nontraumatic subarachnoid hemorrhage from unspecified intracranial artery | 508(2.12) | 87(1.17) | <0.001 | 91(1.22) | 87(1.17) | 0.763 |
| I60.8 Other nontraumatic subarachnoid hemorrhage | 364(1.52) | 54(0.73) | <0.001 | 53(0.71) | 54(0.73) | 0.923 |
| I60.9 Nontraumatic subarachnoid hemorrhage, unspecified | 1915(7.98) | 360(4.84) | <0.001 | 358(4.81) | 360(4.84) | 0.939 |
| I61.0 Nontraumatic intracerebral hemorrhage in hemisphere, subcortical | 1654(6.9) | 560(7.52) | 0.064 | 550(7.39) | 560(7.52) | 0.755 |
| I61.1 Nontraumatic intracerebral hemorrhage in hemisphere, cortical | 1126(4.69) | 302(4.06) | 0.021 | 326(4.38) | 302(4.06) | 0.328 |
| I61.2 Nontraumatic intracerebral hemorrhage in hemisphere, unspecified | 547(2.28) | 174(2.34) | 0.773 | 150(2.02) | 174(2.34) | 0.178 |
| I61.3 Nontraumatic intracerebral hemorrhage in brain stem | 399(1.66) | 136(1.83) | 0.340 | 127(1.71) | 136(1.83) | 0.576 |
| I61.4 Nontraumatic intracerebral hemorrhage in cerebellum | 754(3.14) | 263(3.53) | 0.097 | 252(3.39) | 263(3.53) | 0.622 |
| I61.5 Nontraumatic intracerebral hemorrhage, intraventricular | 1188(4.95) | 440(5.91) | 0.001 | 413(5.55) | 440(5.91) | 0.341 |
| I61.6 Nontraumatic intracerebral hemorrhage, multiple localized | 487(2.03) | 143(1.92) | 0.558 | 146(1.96) | 143(1.92) | 0.859 |
| I61.8 Other nontraumatic intracerebral hemorrhage | 2245(9.36) | 750(10.08) | 0.066 | 752(10.1) | 750(10.08) | 0.957 |
| I61.9 Nontraumatic intracerebral hemorrhage, unspecified | 4825(20.12) | 1573(21.14) | 0.057 | 1656(22.25) | 1573(21.14) | 0.099 |
| I62.0 Nontraumatic subdural hemorrhage | 5598(23.34) | 2056(27.63) | <0.001 | 2016(27.09) | 2056(27.63) | 0.462 |
| I62.1 Nontraumatic extradural hemorrhage | 83(0.35) | 22(0.3) | 0.510 | 15(0.2) | 22(0.3) | 0.394 |
| I62.9 Nontraumatic intracranial hemorrhage, unspecified | 1241(5.17) | 396(5.32) | 0.619 | 441(5.93) | 396(5.32) | 0.876 |

PSM: Propensity Score Matching. T2DM: Type 2 diabetes mellitus.

Supplementary Table 5. International Classification of Disease, Tenth Revision (ICD-10) codes before and after propensity score matching in women patients hospitalized with hemorrhagic stroke as primary diagnosis according to the presence of type 2 diabetes.

|  | BEFORE PSM | | | AFTER PSM | | |
| --- | --- | --- | --- | --- | --- | --- |
|  | No T2DM | T2DM | p-value | No T2DM | T2DM | p-value |
| I60.0 Nontraumatic subarachnoid hemorrhage from carotid siphon and bifurcation | 155(0.76) | 11(0.25) | <0.001 | 9(0.2) | 11(0.25) | 0.654 |
| I60.1 Nontraumatic subarachnoid hemorrhage from middle cerebral artery | 497(2.42) | 30(0.67) | <0.001 | 35(0.78) | 30(0.67) | 0.534 |
| I60.2 Nontraumatic subarachnoid hemorrhage from anterior communicating artery | 530(2.59) | 29(0.65) | <0.001 | 42(0.94) | 29(0.65) | 0.121 |
| I60.3 Nontraumatic subarachnoid hemorrhage from posterior communicating artery | 369(1.8) | 26(0.58) | <0.001 | 20(0.45) | 26(0.58) | 0.375 |
| I60.4 Nontraumatic subarachnoid hemorrhage from basilar artery | 74(0.36) | 4(0.09) | 0.003 | 6(0.13) | 4(0.09) | 0.527 |
| I60.5 Nontraumatic subarachnoid hemorrhage from vertebral artery | 53(0.26) | 0(0) | 0.001 | 4(0.09) | 0(0) | 0.045 |
| I60.6 Nontraumatic subarachnoid hemorrhage from other intracranial arteries | 279(1.36) | 24(0.54) | <0.001 | 20(0.45) | 24(0.54) | 0.546 |
| I60.7 Nontraumatic subarachnoid hemorrhage from unspecified intracranial artery | 758(3.7) | 95(2.12) | <0.001 | 87(1.95) | 95(2.12) | 0.549 |
| I60.8 Other nontraumatic subarachnoid hemorrhage | 440(2.15) | 43(0.96) | <0.001 | 42(0.94) | 43(0.96) | 0.913 |
| I60.9 Nontraumatic subarachnoid hemorrhage, unspecified | 2448(11.94) | 331(7.4) | <0.001 | 342(7.65) | 331(7.4) | 0.659 |
| I61.0 Nontraumatic intracerebral hemorrhage in hemisphere, subcortical | 1083(5.28) | 295(6.6) | <0.001 | 262(5.86) | 295(6.6) | 0.149 |
| I61.1 Nontraumatic intracerebral hemorrhage in hemisphere, cortical | 1068(5.21) | 204(4.56) | 0.074 | 205(4.58) | 204(4.56) | 0.960 |
| I61.2 Nontraumatic intracerebral hemorrhage in hemisphere, unspecified | 550(2.68) | 109(2.44) | 0.353 | 109(2.44) | 109(2.44) | 0.999 |
| I61.3 Nontraumatic intracerebral hemorrhage in brain stem | 268(1.31) | 84(1.88) | 0.003 | 57(1.27) | 84(1.88) | 0.022 |
| I61.4 Nontraumatic intracerebral hemorrhage in cerebellum | 667(3.25) | 207(4.63) | <0.001 | 154(3.44) | 207(4.63) | 0.004 |
| I61.5 Nontraumatic intracerebral hemorrhage, intraventricular | 975(4.76) | 246(5.5) | 0.037 | 239(5.34) | 246(5.5) | 0.744 |
| I61.6 Nontraumatic intracerebral hemorrhage, multiple localized | 362(1.77) | 89(1.99) | 0.308 | 90(2.01) | 89(1.99) | 0.940 |
| I61.8 Other nontraumatic intracerebral hemorrhage | 1737(8.47) | 430(9.61) | 0.014 | 451(10.08) | 430(9.61) | 0.456 |
| I61.9 Nontraumatic intracerebral hemorrhage, unspecified | 4204(20.51) | 1001(22.38) | 0.005 | 1104(24.68) | 1001(22.38) | 0.010 |
| I62.0 Nontraumatic subdural hemorrhage | 2907(14.18) | 922(20.61) | <0.001 | 888(19.85) | 922(20.61) | 0.371 |
| I62.1 Nontraumatic extradural hemorrhage | 74(0.36) | 20(0.45) | 0.394 | 21(0.47) | 20(0.45) | 0.876 |
| I62.9 Nontraumatic intracranial hemorrhage, unspecified | 1004(4.9) | 273(6.1) | 0.001 | 286(6.39) | 273(6.1) | 0.570 |

PSM: Propensity Score Matching. T2DM: Type 2 diabetes mellitus.
